# A Study on Symbiotic Systems of Cicadas Provides New Insights into Distribution of Microbial Symbionts and Improves Fluorescence In Situ Hybridization Technique

**DOI:** 10.3390/ijms24032434

**Published:** 2023-01-26

**Authors:** Zhi Huang, Jinrui Zhou, Zhijun Zhang, Hong He, Cong Wei

**Affiliations:** 1 Key Laboratory of Plant Protection Resources and Pest Management of the Ministry of Education, College of Plant Protection, Northwest A&F University, Xianyang 712100, China; 2 College of Forestry, Northwest A&F University, Xianyang 712100, China

**Keywords:** symbiosis, sap-feeding insects, *Sulcia*, YLS, evolution

## Abstract

Nutritional symbionts of sap-sucking auchenorrhynchan insects of Hemiptera are usually confined to the bacteriomes and/or fat bodies. Knowledge is limited about the distribution of microbial symbionts in other organs. We investigated the distribution of obligate symbionts in the salivary glands, gut tissues, reproductive organs, bacteriomes, and fat bodies of two cicada species, *Karenia caelatata* and *Tanna* sp., using integrated methods, including a modified fluorescence in situ hybridization (FISH) technique, which can greatly enhance the FISH signal intensity of related symbionts. We revealed that *Candidatus* Sulcia muelleri (*Sulcia*) and a yeast-like fungal symbiont (YLS) were harbored in the bacteriomes and fat bodies, respectively. Both of *Sulcia* and YLS can be transmitted to the offspring via ovaries, forming a “symbiont ball” in each egg. Neither *Sulcia* nor YLS were harbored in the salivary glands, gut tissues and testes. Phylogenetic trees of both *Sulcia* and cicadas confirm that *K. caelatata* is a member of the tribe Dundubiini, and the tribe Leptopsaltriini that comprises *Ta.* sp. is not monophyletic. YLS of *K. caelatata* is embedded inside the lineage of YLS of Dundubiini, whereas YLS of *Ta.* sp. is closely related to the clade comprising both cicada-parasitizing fungi *Ophiocordyceps* and YLS of *Mogannia conica* and *Meimuna mongolica*, suggesting an evolutionary replacement of YLS in *Ta.* sp. from an *Ophiocordyceps* fungus to another *Ophiocordyceps* fungus. Our results provide new insights into the symbiosis between Cicadidae and related symbionts. Modification through the addition of helpers and heat shock greatly enhanced the FISH signal intensity of YLS, which may provide guidelines for enhancement of the hybridization signal intensity of other symbiont(s) in the FISH experiments.

## 1. Introduction

The symbiosis between host insects and symbionts are quite common in nature, especially for the sap-feeding groups and their microbial symbionts [1,2,3]. The sap-sucking insects of Hemiptera feed exclusively on the plant xylem or phloem sap, which is nutritionally unbalanced in vitamins and essential amino acids [4]. Most sap-feeding insects usually harbor nutritional symbionts in a highly specialized organ called bacteriomes or mycetomes and are obligately dependent on these symbionts for the provision of essential amino acids and other nutrients that are deficient in the plant sap [4,5,6].

The most extraordinary systems of symbiosis in insects are found in the plant sap-feeding insects in the suborder Auchenorrhyncha of Hemiptera, including spittlebugs, leafhoppers, treehoppers, cicadas, and planthoppers [7]. The ancestor of Auchenorrhyncha established an intimate symbiosis with a Bacteroidetes (*Candidatus* Sulcia muelleri (hereafter referred to as *Sulcia*) approximately 260 million years ago) and a betaproteobacterium [7]. The betaproteobacterium was still retained in some sap-feeding groups, whereas it was replaced by other bacteria or even fungi in other groups over evolutionary time [8,9,10]. The proteobacterium varied in different sap-feeding groups, e.g., *Candidatus* Zinderia insecticola in some spittlebugs [11,12], *Candidatus* Nasuia deltocephalinicola in some leafhoppers and treehoppers [13,14], *Candidatus* Vidania fulgroidaea (hereafter referred to as *Vidania*) in some planthoppers [15,16], and *Candidatus* Hodgkinia cicadicola (hereafter referred to as *Hodgkinia*) in some cicadas [10,17]. In addition to *Sulcia* and the coresident symbiont(s), the auchenorrhynchan insects have been reported to be associated with some secondary symbionts, including *Wolbachia*, *Sodalis*, *Spiroplasma*, *Rickettsia*, *Serratia,* and *Arsenophonus* [10,14,16,17]. It has been reported that *Sulcia* and *Vidania* provide host insects with essential amino acids, whereas *Sodalis*, *Wolbachia,* and *Arsenophonus* can participate in the synthesis of essential amino acids and B vitamins in planthoppers *Callodictya krueperi*, *Dictyophara multireticulata,* and *Ranissus scytha*, respectively [16].

Cicadas feed exclusively on nutritionally unbalanced xylem sap and have established an intimate symbiosis with the obligate symbionts *Sulcia* and *Hodgkinia* for the provision of essential amino acids, cobalamin, and vitamins [18,19,20]. The *Sulcia* genome possesses the biosynthetic pathways for eight of the ten essential amino acids, whereas the *Hodgkinia* genome is complementarily retained for the synthesis of the remaining two essential amino acids (i.e., methionine and histidine), cobalamin, and vitamins for the host cicadas [20]. In some species of the genera *Magicicada* and *Tettigades*, *Hodgkinia* has evolved into two or more complex cytologically distinct but metabolically interdependent cellular lineages [21,22,23]. In the majority of *Hodgkinia*-free cicada species, a yeast-like fungal symbiont (hereafter referred to as YLS) is harbored in the fat bodies, whereas it is harbored in both the bacteriome sheath and fat bodies in a few species, e.g., *Hyalessa maculaticollis* and *Graptopsaltria tienta* [9,10]. The molecular phylogenetic analysis uncovered that YLS of cicadas form a clade within the entomoparasitic fungi of the genus *Ophiocordyceps*, which is regarded as a beneficial symbiont evolved from a pathogenic microorganism [9]. Recently, it was revealed that there were five independent replacement events in the loss of *Hodgkinia*/acquisition of YLS and seven other separate replacement events of YLS (from an *Ophiocordyceps* fungus to another *Ophiocordyceps* fungus) within the sampled taxa of Cicadidae [24]. Genome sequencing of YLS of *Meimuna opalifera* revealed that the genome of YLS encodes biosynthetic pathways for synthesizing almost all essential amino acids and other nutrients, which is sufficient to compensate for the nutritional services provided by *Hodgkinia* [9]. 

The phylogeny of *Sulcia* generally mirrors that of host insects due to the strictly transovarial transmission of this symbiont [7,25]. Recently, a new subfamily Derotettiginae was established for the Cicadidae based on phylogenetic analyses of both cicadas and their symbiont *Sulcia* [26]. By contrast, the phylogeny of YLS and related *Ophiocordyceps* is generally unparallel to that of host insects, but the phylogeny of partial YLS lineages is concordant with that of host insects [9,24]. Therefore, the phylogeny of *Sulcia* and that of YLS, as well as related *Ophiocordyceps,* may provide additional molecular evidence to clarify the coevolutionary history of Cicadidae and the symbionts. However, such symbionts in many Cicadidae taxa with the phylogenetic status remaining questionable have not been investigated. To date, a few microscopic studies have been conducted on the distribution of obligate symbionts in the digestive and reproductive organs, bacteriomes, and fat bodies of some cicada species. A previous study based on diagnostic PCR amplification briefly reported that *Sulcia* can be detected from the filter chamber and the conical segment of cicadas *Platypleura kaempferi* and *Me. mongolica* [27]. Another study based on Illumina genome sequencing and fluorescence in situ hybridization (FISH) microscopy reported that *Sulcia* distributes in the digestive and excretory organs beside the bacteriomes and gonads in cicada *Subpsaltria yangi* [28]. However, most previous studies have shown that *Sulcia* is harbored in the bacteriomes, which can be transovarially transmitted to the offspring in auchenorrhynchan insects [9,10,17]. *Sulcia* harbored in other tissues besides the bacteriomes and ovaries in cicadas merits further investigation of more species based on integrated methods, including molecular sequencing, histological, and fluorescence microscopy. 

In this study, we initially investigated the microbial communities associated with the bacteriomes, fat bodies, and reproductive organs (i.e., testes and ovaries) of two mountain-habitat specialist cicada species, *Tanna* sp. and *Karenia caelatata*, based on Illumina high-throughput sequencing. Diagnostic PCR amplification was also performed to explore the presence of obligate symbionts in the salivary glands; gut tissues (i.e., filter chamber, conical segment, midgut, and hindgut); reproductive organs (i.e., testes and ovaries); fat bodies; and bacteriomes of these two cicadas. The phylogenetic trees were reconstructed based on these two cicada species and other representative cicada species, as well as their obligate symbionts, aiming to resolve the phylogenetic affiliation of the host cicadas and their symbionts. We further explored the distribution of obligate symbionts in the salivary glands, gut tissues, reproductive organs, bacteriomes, and fat bodies using histological and fluorescence microscopy. Additionally, we compared the fluorescence signal intensity of related symbionts based on different treatments, aiming to explore the optimized methods for improving the fluorescence signal intensity of symbionts in the fluorescence in situ hybridization (FISH) experiments. As a result, the modified FISH technique by adding unlabeled helper sequences and heat shock can significantly enhance the fluorescence signal intensity of YLS, which may provide new insights into the enhancement of hybridization signal intensity of other symbiont(s) in the FISH experiments.

## 2. Results

### 2.1. Microbial Composition in the Reproductive Organs, Fat Bodies, and Bacteriomes of K. caelatata and Ta. sp.

**Figure 1:**
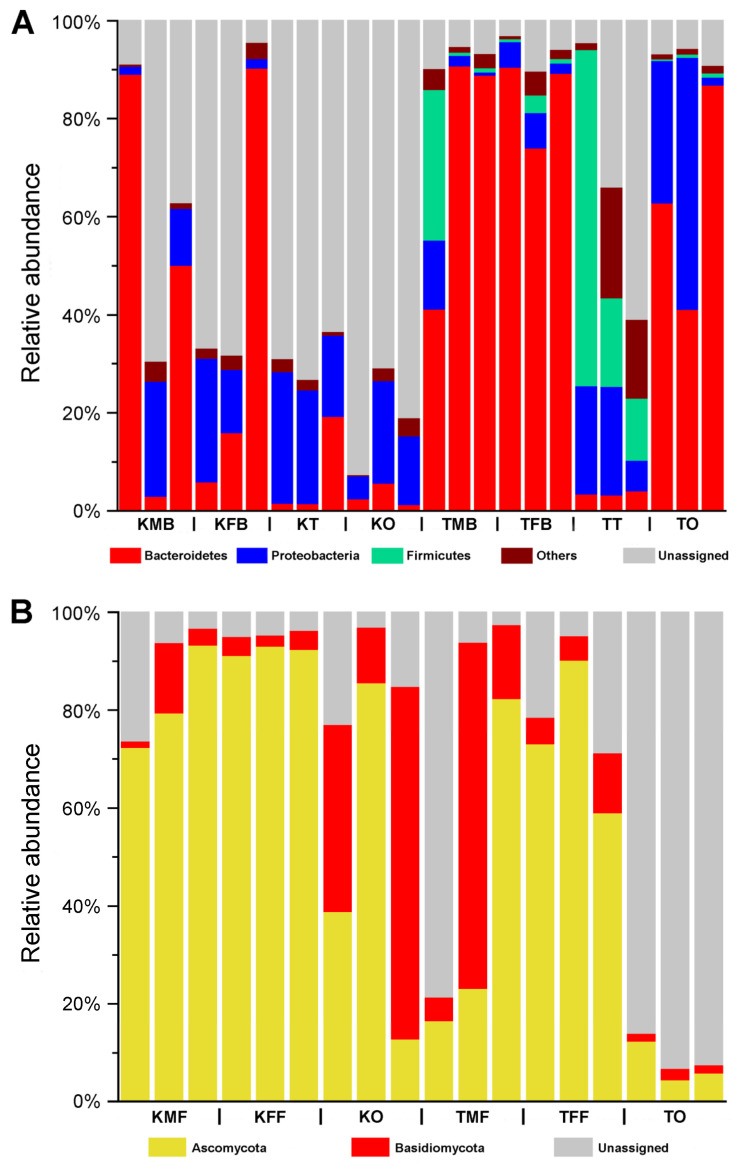
Microbial communities in the reproductive organs, fat bodies, and bacteriomes of *Karenia caelatata* and *Tanna* sp. at the phylum level. (**A**) Bacterial communities in the reproductive organs and bacteriomes of *K. caelatata* and *Ta.* sp. at the phylum level. (**B**) Fungal communities in the ovaries and fat bodies of *K. caelatata* and *Ta.* sp. at the phylum level. Abbreviations: KFB, the bacteriomes of *K. caelatata* females; KFF, the fat bodies of *K. caelatata* females; KMB, the bacteriomes of *K. caelatata* males; KMF, the fat bodies of *K. caelatata* males; KO, the ovaries of *K. caelatata* females; KT, the testes of *K. caelatata* males; TFB, the bacteriomes of *Ta*. sp. females; TFF, the fat bodies of *Ta*. sp. females; TMB, the bacteriomes of *Ta*. sp. males; TMF, the fat bodies of *Ta*. sp. males; TO, the ovaries of *Ta*. sp. females; TT, the testes of *Ta*. sp. males.

We categorized the assigned phyla or genera with relatively low abundances as “others” and the unassigned sequences as “unassigned”. The “others” represented the assigned phyla or genera with an abundance of <2% across all samples. The identified sequences were mainly belonged to three bacterial phyla and two fungal phyla, respectively. At the bacterial phylum level, the most common bacterial phyla were Bacteroidetes and Proteobacteria among all the samples of *K. caelatata* and *Ta*. sp. (Figure 1A). At the fungal phylum level, Ascomycota was the most dominant phylum in the bacteriomes and ovaries of these two cicada species, followed by Basidiomycota (Figure 1B).

**Figure 2:**
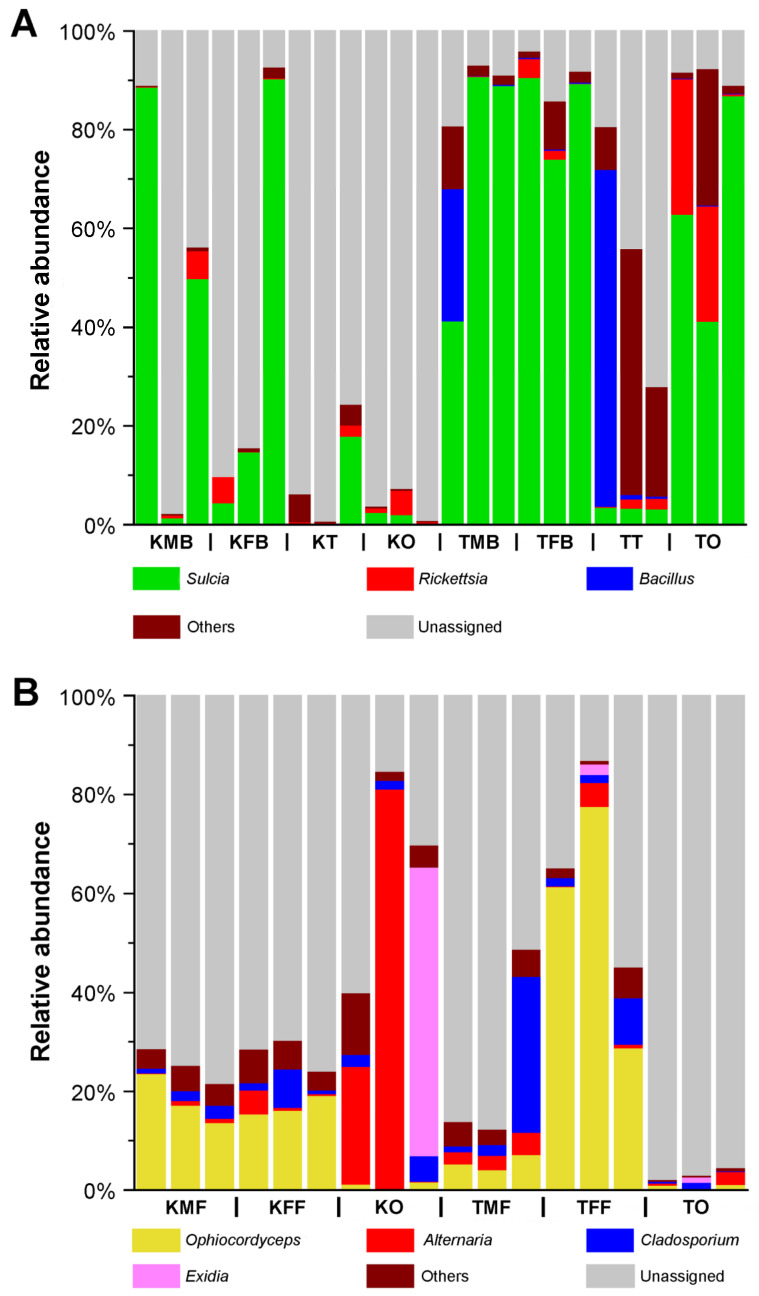
Microbial communities in the reproductive organs and bacteriomes of *Karenia caelatata* and *Tanna* sp. at the genus level. (**A**) Bacterial communities in the reproductive organs and bacteriomes of *K. caelatata* and *Ta.* sp. at the genus level. (**B**) Fungal communities in the ovaries and fat bodies of *K. caelatata* and *Ta.* sp. at the genus level. Abbreviations are the same as those in Figure 1.

At the bacterial genus level, *Sulcia* accounted for relatively high proportions in the bacteriomes (males, 1.41 to 88.65%; females, 4.37 to 90.31%) of *K. caelatata*, but it exhibited a relatively low abundance in the ovaries (0.33 to 2.45%) (Figure 2A). In contrast, *Sulcia* was prominent in the bacteriomes (males, 41.22 to 90.76%; females, 73.99 to 90.56%) and the ovaries (41.15 to 86.88%) of *Ta*. sp., but it exhibited an extremely low abundance in the testes (3.16 to 3.49%). The relative abundance of *Rickettsia* varied considerably across all the samples. Additionally, *Bacillus* was the dominant genus in the bacteriomes (26.64%) and testes (67.56%) of one male of *Ta*. sp., and it exhibited a significantly low abundance in the other samples of both cicada species (Figure 2A).

At the fungal genus level, *Ophiocordyceps* was the dominant genus in the fat bodies of both sexes (*K. caelatata*, 13.75 to 23.65%; *Ta*. sp., 4.11 to 77.67%), but it exhibited a low abundance in the ovaries (*K. caelatata*, 0.23 to 1.70%; *Ta.* sp., 0.16 to 1.19%) of these two species (Figure 2B). The relative abundance of the remaining genera (i.e., *Alternaria*, *Cladosporium* and *Exidia*) varied considerably among different samples (Figure 2B).

### 2.2. Diagnostic Pcr of Dominant Symbionts Obtained from K. caelatata and Ta. Sp.

The results of the diagnostic PCR amplification revealed that *Sulcia* was detected in the bacteriomes and the ovaries of both *Ta*. sp. and *K. caelatata*, whereas YLS was detected in the fat bodies and the ovaries. Neither *Sulcia* nor YLS were detected in the salivary glands, filter chamber, conical segment, midgut, and the hindgut of these two species (Table S1). *Sulcia* of *Ta*. sp. exhibited an extremely high similarity to that of *Ta*. *japonensis* (99.40%), *Terpnosia vacua* (98.96%), and *Te. Nigricosta* (98.81%), whereas YLS of *Ta*. sp. exhibited the highest similarity to that of *Mogannia minuta* (95.38%). *Sulcia* of *K. caelatata* showed the highest similarity to that of *Me. iwasakii* (99.93%), *Me. kuroiwae* (99.93%), and *Me. oshimensis* (99.85%), and YLS of *K. caelatata* showed a significantly high similarity to that of *Me. oshimensis* (92.95%) and *Me. opalifera* (90.78%) (Table S2). 

### 2.3. Distribution of Sulcia and YLS in Salivary Glands, Gut Tissues, Reproductive Organs, Bacteriomes, and Fat Bodies

**Figure 3:**
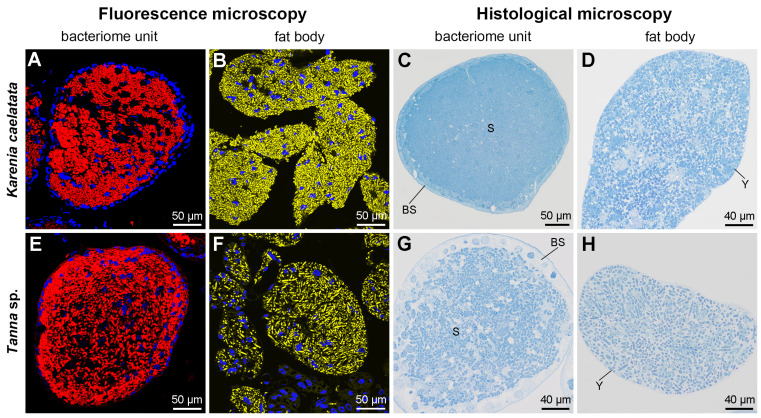
Histological and fluorescence microscopy showing the distribution of *Sulcia* and YLS in the bacteriomes and fat bodies of *Karenia caelatata* and *Tanna* sp. (**A**–**D**) Distribution of *Sulcia* and YLS in the bacteriomes and fat bodies of *K. caelatata.* (**E**–**H**) Distribution of *Sulcia* and YLS in the bacteriomes and fat bodies of *Ta*. sp. For fluorescence microscopy, blue, yellow, and red represent the nucleus, YLS, and *Sulcia*, respectively. Abbreviations: BS, bacteriome sheath; S, *Sulcia*; Y, yeast-like fungal symbiont.

**Figure 4:**
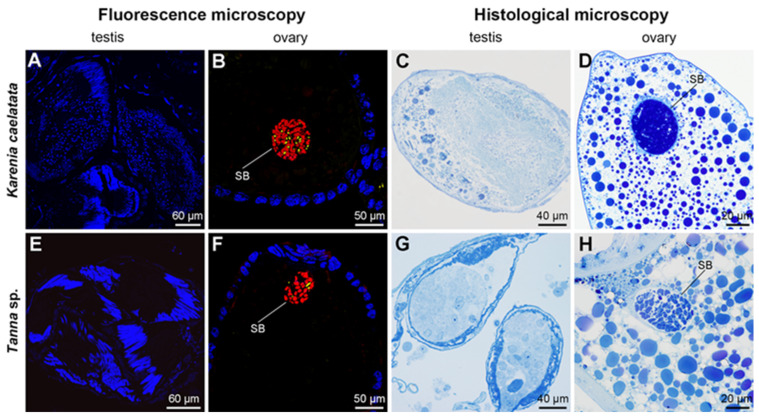
Histological and fluorescence microscopy showing the distribution of *Sulcia* and YLS in the testes and ovaries of *Karenia caelatata* and *Tanna* sp. (**A**–**D**) Distribution of *Sulcia* and YLS in the testes and ovaries of *K. caelatata.* (**E**–**H**) Distribution of *Sulcia* and YLS in the testes and ovaries of *Ta*. sp. For fluorescence microscopy, blue, yellow, and red represent the nucleus, YLS, and *Sulcia*, respectively. Abbreviations: SB, symbiont ball.

**Figure 5:**
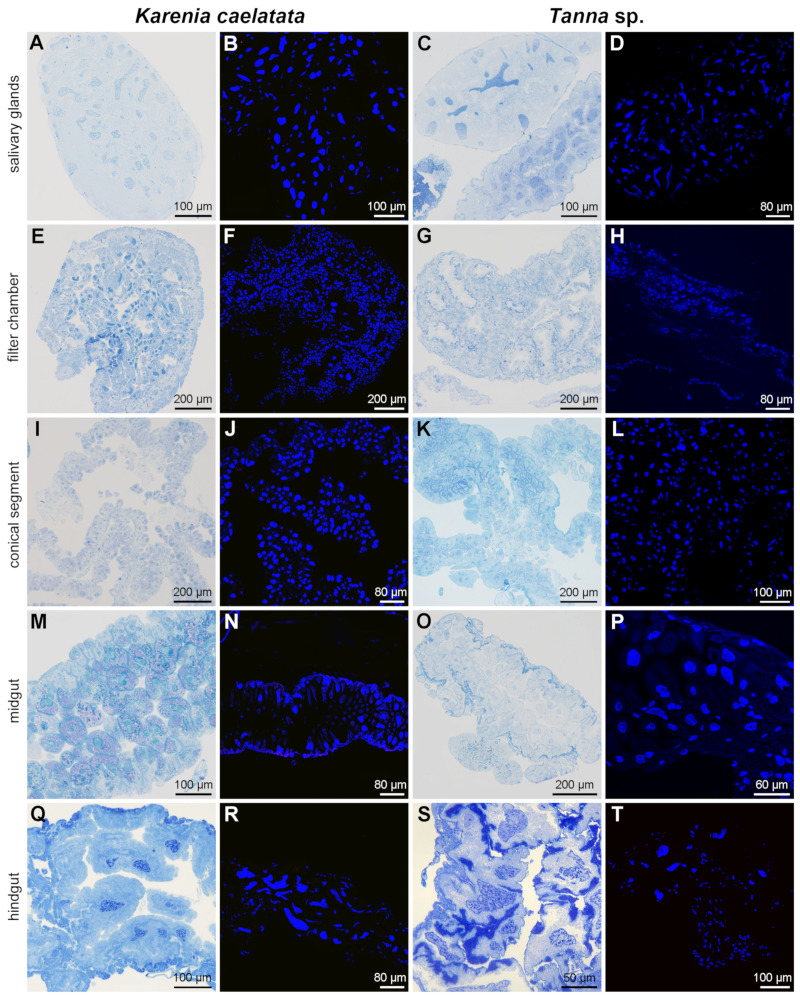
Histological and fluorescence microscopy showing the distribution of *Sulcia* and YLS in the salivary glands, filter chamber, conical segment, midgut, and hindgut of *Karenia caelatata* and *Tanna* sp. (**A**,**B**) Distribution of *Sulcia* and YLS in the salivary glands of *K. caelatata.* (**C**,**D**) Distribution of *Sulcia* and YLS in the salivary glands of *Ta*. sp. (**E**,**F**) Distribution of *Sulcia* and YLS in the filter chamber of *K. caelatata.* (**G**,**H**) Distribution of *Sulcia* and YLS in the filter chamber of *Ta*. sp. (**I**,**J**) Distribution of *Sulcia* and YLS in the conical segment of *K. caelatata.* (**K**,**L**) Distribution of *Sulcia* and YLS in the conical segment of *Ta*. sp. (**M**,**N**) Distribution of *Sulcia* and YLS in the midgut of *K. caelatata.* (**O**,**P**) Distribution of *Sulcia* and YLS in the midgut of *Ta*. sp. (**Q**,**R**) Distribution of *Sulcia* and YLS in the hindgut of *K. caelatata.* (**S**,**T**) Distribution of *Sulcia* and YLS in the hindgut of *Ta*. sp. For fluorescence microscopy, blue represent the nucleus.

Histological and fluorescent microscopy showed that *Sulcia* was harbored in the bacteriomes of *K. caelatata* and *Ta*. sp., while YLS occupied the fat bodies of these two species (Figure 3 and Figure S1). YLS and *Sulcia* formed a “symbiont ball” in mature oocytes of the ovaries, whereas both of them were not found in the testes of these two species (Figure 4 and Figure S1). Neither *Sulcia* nor YLS were harbored in the bacteriome sheath of these two cicada species (Figure 3 and Figure S1). Both YLS and *Sulcia* were not found in the salivary glands, filter chamber, conical segment, midgut, and the hindgut of *K. caelatata* and *Ta*. sp. (Figure 5).

**Figure 6:**
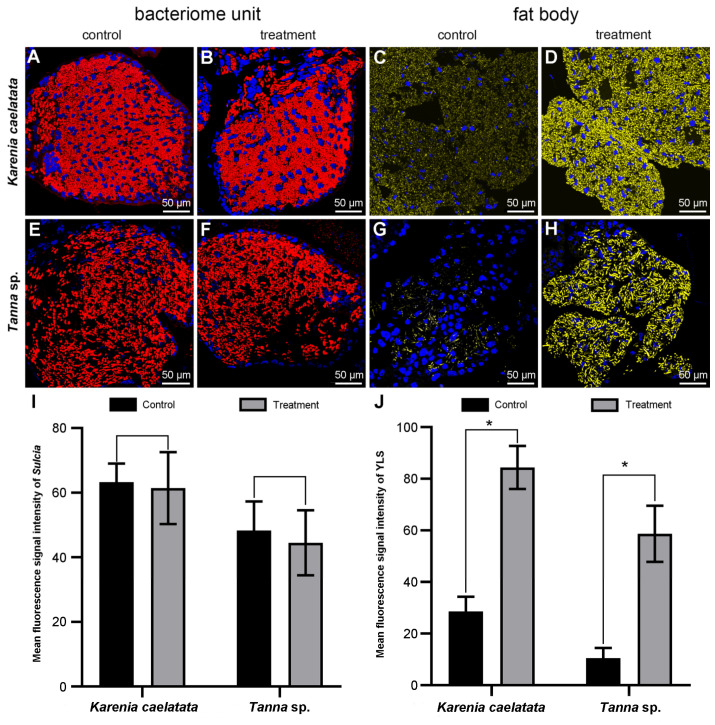
Fluorescence microscopy showing the fluorescence signal intensity of *Sulcia* and YLS based on different treatments for *Karenia caelatata* and *Tanna* sp. (**A**,**B**) *Sulcia* in the bacteriomes of *K. caelatata.* (**C**,**D**) YLS in the fat bodies of *K. caelatata.* (**E**,**F**) *Sulcia* in the bacteriomes of *Ta.* sp. (**G**,**H**) YLS in the fat bodies of *Ta.* sp. (**I**) Compared analysis of the fluorescence signal intensity of *Sulcia* between control groups and treatment groups. (**J**) Compared analysis of the fluorescence signal intensity of YLS between control groups and treatment groups. Control, without addition of helpers and heat shock. Treatment, with addition of helpers into hybridization buffer combined with 95 °C heat shock of hybridization sections for 2 min before hybridization. Asterisks (“*”) representing the significant differences between control groups and treatment groups.

For *K. caelatata* and *Ta*. sp., the fluorescence signal intensity of *Sulcia* was generally similar for the two different treatments. The addition of unlabeled helper sequences and 95 °C heat shock had no obvious effect on the enhancement of the *Sulcia* signal intensity when compared with the fluorescence signal intensity of *Sulcia* under the control experiment (viz., without the addition of helpers and heat shock) (Figure 6). For both cicada species, the fluorescence signal intensity of YLS was significantly enhanced by the addition of helpers into a hybridization buffer combined with 95 °C heat shock of the hybridization sections for 2 min before hybridization when compared with the fluorescence signal intensity of YLS under the control experiment (viz., without addition of unlabeled helper sequences and 95 °C heat shock). The YLS fluorescence signal intensity of the control groups/treatment groups were approximately 1:2.95 and 1:5.58 in *K. caelatata* and *Ta*. sp., respectively (Figure 6). These results clearly showed that the fluorescence signal intensity of YLS can be greatly enhanced by the addition of unlabeled helper sequences and 95 °C heat shock, whereas it has not obvious enhancement of the signal intensity of *Sulcia*.

### 2.4. Phylogenetic Relationships of Sulcia and YLS in Cicadas

**Figure 7:**
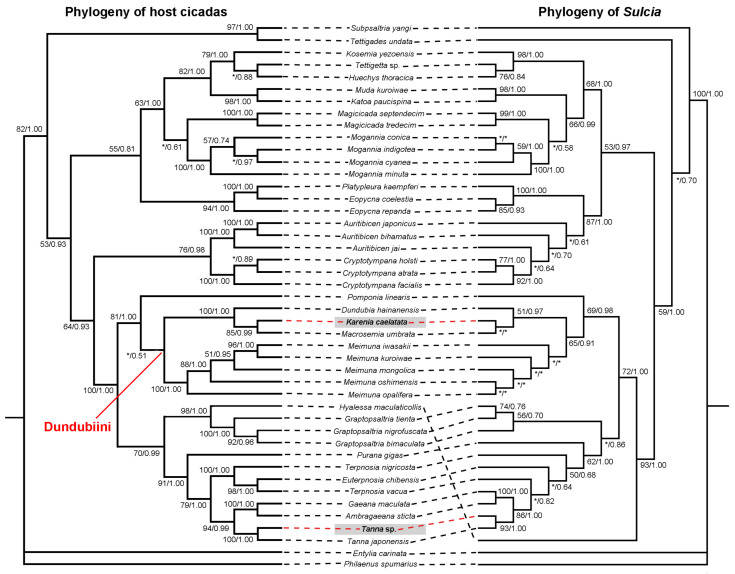
Co-phylogeny of host cicadas and *Sulcia*. The phylogenetic relationship of host cicadas was reconstructed based on joint mitochondrial genes (*COI* + *COII* + *Cytb*). The phylogenetic relationship of *Sulcia* was reconstructed based on 16S rRNA gene sequences. Asterisks (“*”) representing support values less than 50% and bootstrap support values more than 50% are shown on each node in the order of the maximum-likelihood/Bayesian inference. Bootstrap support values and posterior probabilities of the maximum-likelihood/Bayesian inference are shown near branches.

The phylogenetic trees of both *Sulcia* and host cicadas show that the phylogeny of *Sulcia* is generally congruent with the phylogeny of host cicadas (Figure 7). The phylogenetic tree of host cicadas show that *K. caelatata* is embedded inside the tribe Dundubiini of the subfamily Cicadinae, which is closely related to *Macrosemia umbrata* and *Dundubia hainanensis* (Figure 7). Correspondingly, the phylogeny of *Sulcia* shows that *Sulcia* of *K. caelatata* is embedded inside the lineage comprising *Sulcia* of Dundubiini (Figure 7). The phylogenetic trees of both *Sulcia* and host cicadas showed that *Ta*. sp. is closely related to *Ta. japonensis* (Figure 7). Specifically, *Ta.* sp. and *Ta. japonensis* form a sister group in the lineage that includes species of the tribe Gaeanini, i.e., *Gaeana maculate* and *Ambragaeana sticta*, whereas *Ga. maculate* and *A. sticta* are embedded inside the tribe Leptopsaltriini (Figure 7).

**Figure 8:**
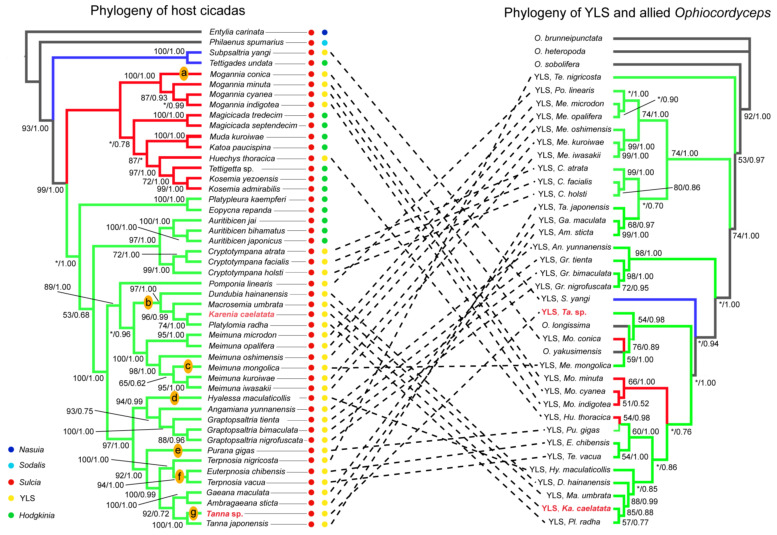
Compared analysis of the phylogeny of host cicadas and YLS. The phylogenetic relationship of host cicadas was reconstructed based on joint mitochondrial genes (*COI* + *COII* + *Cytb*). The phylogenetic relationship of YLS was reconstructed based on joint mitochondrial genes (*18S* + *28S* + *RPB1* + *RPB2* + *EF-1α*). Letters “a–g” on the phylogeny representing the replacements of YLS (from an *Ophiocordyceps* fungus to another *Ophiocordyceps* fungus). Asterisks (“*”) representing support values less than 50% and bootstrap support values more than 50% are shown on each node in the order of the maximum-likelihood/Bayesian inference. Bootstrap support values and posterior probabilities of the maximum-likelihood/Bayesian inference are shown near branches.

Phylogenetic trees of YLS and allied *Ophiocordyceps* compared to the trees of representative cicada species show that the phylogeny of YLS is largely incongruent with the phylogeny of host cicadas, although the phylogeny of some YLS lineages (e.g., *Graptopsaltria* species) is partially concordant with that of related cicada lineages (Figure 8). Phylogenetic trees of cicada fungal symbionts and cicada-parasitizing fungi show that some cicada-parasitizing fungi (i.e., *Ophiocordyceps longissima* and *O. yakusimensis*) are placed within the clade of cicada symbionts, whereas others (e.g., *O. sobolifera*) are placed outside the clade of these symbionts (Figure 8). The phylogenetic trees of both YLS and host cicadas show that *K. caelatata* is closely related to *Platylomia radha*, *Ma. Umbrata,* and *D. hainanensis* (Figure 8). Phylogenetic trees of YLS and allied *Ophiocordyceps* show that YLS of *Ta*. sp. is embedded inside the clade, including cicada-parasitizing fungi *O. longissima* and *O. yakusimensis*. However, the trees of host cicadas show that *Ta*. sp. is closely related to *Ta. japonensis* (Figure 8). Additionally, our results reveal seven replacement events of YLS, from an *Ophiocordyceps* fungus to another *Ophiocordyceps* fungus, such as the YLS of *Ta*. sp. and *K. caelatata* (Figure 8).

## 3. Discussion

### 3.1. Distribution of Symbionts in Different Tissues of Cicadas 

Our results revealed that *Hodgkinia* is absent and YLS is present in *K. caelatata* and *Ta*. sp. (Figure 2 and Tables S1 and S2). *Sulcia* was harbored in the bacteriomes, while YLS occupied the fat bodies in these two cicada species (Figure 3 and Figure S1). Both *Sulcia* and YLS can be transovarially transmitted between generations of the host cicada, which formed a “symbiont ball” in the mature oocytes of the ovaries (Figure 4 and Figure S1). Based on the results of Illumina sequencing, *Sulcia* showed a relatively low abundance in the ovaries of *K. caelatata* (0.33 to 2.45%) (Figure 2A), and *Ophiocordyceps* (YLS) exhibited a low abundance in the ovaries of both cicada species (*K. caelatata*, 0.23 to 1.70%; *Ta*. sp., 0.16 to 1.19%) (Figure 2B). A reasonable explanation may be that the relative abundance of *Sulcia* and YLS in the ovaries of these cicadas could be closely related to the number of the symbionts transmitted to the ovaries. The results of histological and fluorescence microscopy also revealed that *Sulcia* was not harbored in the testes, although it exhibited a low abundance in some samples of testes in these two cicadas (Figure 2A, Figure 4 and Figure S1). We hypothesized that *Sulcia* in the hemolymph can adhere to the surface of testes, resulting in a low abundance of *Sulcia* that was detected in some samples of testes through Illumina high-throughput sequencing. 

Previous studies reported that *Sulcia* can be detected in the filter chamber and conical segment of cicadas *Platyp. kaempferi* and *Me. mongolica* and in the digestive and excretory organs of cicada *Su. yangi* [27,28]. However, our results revealed that neither *Sulcia* nor YLS were harbored in the salivary glands and gut tissues in the two sampled cicada species (Figure 5 and Table S1). One explanation seems to be that the distribution of *Sulcia* is distinct for different species. However, an alternative explanation is that *Sulcia* in the hemolymph can also adhere to the surfaces of some tissues, including the gut tissues (e.g., filter chamber and conical segment), resulting in the detection of *Sulcia* in these tissues, except for the bacteriomes and ovaries based on the Illumina high-throughput sequencing and/or diagnostic PCR amplification. The distribution of *Sulcia* in most sampled cicada species was only investigated based on diagnostic PCR amplification or Illumina sequencing, which still needs to be further confirmed using other methods such as fluorescence and histological microscopy.

In our present study, *Rickettsia* was detected in the testes, ovaries, and bacteriomes of both cicada species, which exhibited a high relative abundance in the ovaries of two individuals of *Ta*. sp. (Figure 2A). *Alternaria* was detected in the ovaries and fat bodies of both cicada species, but it exhibited a high relative abundance in the ovaries of two individuals of *K. caelatata* (Figure 2B). Previous studies reported that some facultative symbionts, including *Wolbachia*, *Sodalis*, *Spiroplasma*, *Rickettsia*, *Serratia,* and *Arsenophonus*, can provide the hosts with benefits under certain conditions [29,30,31,32,33]. The exact functions and transmission mechanism of *Rickettsia* and *Alternaria* in Cicadidae remain unknown, which need to be clarified in the future. It is possible that the microbial communities in cicadas are more complex than currently appreciated when microbial communities of more cicada species are investigated using the integrated methods. 

### 3.2. Cophylogeny of Sulcia and Host Cicadas Reflecting Host–Symbiont Codiversification

The phylogeny of *Sulcia* could mirror that of host insects due to that this symbiont is strictly transovarially transmitted between generations of host insects [7,25]. The cicada tribe Sinosenini, comprising only the genus *Karenia*, is an unusual lineage in Cicadidae in that the males lack the sound-producing timbal organs, and species of this group are referred to as “mute” cicadas [34]. A previous study based on mitochondrial genomes of 62 cicada species revealed that the mute cicada *K. caelatata* is closely related to representatives of the tribe Dundubiini in the subfamily Cicadinae and concluded that Sinosenini should be transferred from Cicadettinae to Cicadinae [35]. Another study based on three molecular markers of 80 cicada species revealed that the mute cicada *K. ravida* is most closely related to the species of the genus *Macrosemia* of Dundubiini and, therefore, synonymized Sinosenini with Dundubiini [36]. Our results revealed that *Sulcia* of *K. caelatata* showed the highest similarity to that of *Me. iwasakii* (99.93%), *Me. kuroiwae* (99.93%), and *Me. oshimensis* (99.85%) (Table S2). The phylogenetic trees of both host cicadas and *Sulcia* revealed that *K. caelatata* is embedded inside the tribe Dundubiini (Figure 7). Therefore, our results strengthen the mergence of Sinosenini with Dundubiini.

A previous study based on molecular sequences of 80 cicada species revealed that Gaeanini and Leptopsaltriini appear polyphyletic and concluded that these two tribes need further revision based on more detailed morphological features [36]. The phylogenetic trees of both *Sulcia* and host cicadas reconstructed in our present study show that *Ta*. sp. and *Ta. japonensis* form a sister group in the lineage that includes species of the tribe Gaeanini, i.e., *Ga. maculate* and *A. sticta*, whereas *Ga. maculate* and *A. sticta* are embedded inside the tribe Leptopsaltriini (Figure 7). This confirms that Leptopsaltriini is not a monopyphyletic group. Therefore, the phylogenetic relationships among Gaeanini, Leptopsaltriini, and other related taxa need to be further investigated based on more morphological features and molecular data, including both molecular sequences of host cicadas and their symbiont *Sulcia*.

### 3.3. The Phylogeny of YLS and Cicadas Revealing Complex Evolutionary Trajectories of YLS

The phylogenetic trees of both YLS and host cicadas show that *K. caelatata* is closely related to *Platyl. radha*, *Ma. Umbrata,* and *D. hainanensis* (Figure 8). This result is generally concordant with the above observation (Figure 7) that *Sulcia* of *K. caelatata* is closely related to that of *Ma. umbrata* and *D. hainanensis*, which suggests that the phylogeny of *Sulcia* and that of YLS, as well as related *Ophiocordyceps,* may provide additional molecular evidence to clarify the host phylogeny in Cicadidae.

The phylogenetic trees of YLS and allied *Ophiocordyceps* show that YLS of *Ta*. sp. is closely related to the clade comprising cicada-parasitizing fungi *O. longissima* and *O. yakusimensis,* as well as the YLS of *Mo. conica* and *Me. mongolica* (Figure 8). This suggests an evolutionary replacement of YLS in *Ta*. sp. occurred from an *O*. fungus to another *O*. fungus, indicating complex evolutionary trajectories of YLS/*Ophiocordyceps* existed in different host insects. It has been reported that most insects are associated with fungi in interactions that range from pathogenicity to mutualism from the host perspective [27,28,29,30,31,32,33,34,35,36,37,38,39]. Future studies on the phylogeny of YLS and allied *Ophiocordyceps* in cicadas and other sap-feeding insects may provide novel insights into the complex evolutionary processes of YLS and their host insects.

### 3.4. The Improvement of FISH Signal Intensity for Related Symbiont(s)

FISH with rRNA-targeted oligonucleotide probes can be a cultivation-independent method for detecting microorganisms in complex samples, such as animal tissues, clinical specimens, and so on [40]. However, the FISH technique still suffers from some limitations, including the inaccessibility of the probe-binding sites, which may result in the weakness or the absence of specific fluorescence signal intensities of targeted microorganisms. This may eventually lead to the underestimation or misunderstanding of the density and distribution of related microorganisms. It is still necessary to explore the optimized methods for improving the fluorescence signal intensity of some targeted microorganisms for the FISH experiments.

The preservation of the samples in the fixatives is crucial for the success of the FISH experiments. The samples’ preservation in fixatives can preserve the integral morphology of the samples and prevent cell lysis from enzymatic digestion by related proteases. More importantly, it permeabilizes the cell membrane to allow the rRNA-targeted oligonucleotide probes to diffuse easily to intracellular rRNA-targeted sites, which increases the accessibility of the probe target sites. The main fixatives are acetone, ethanol, and 4% paraformaldehyde. Although acetone can deprive water from dissected tissue samples, it easily makes tissues soaked. Therefore, ethanol and 4% paraformaldehyde are still the best choices for the sample fixation [41]. Specifically, we recommend using 100% ethanol for the fixation of fungal symbionts (e.g., YLS) that contain an incrassate cell wall.

Currently, oligonucleotide probes have been commonly used for the detection of related microorganisms, especially for the insect symbionts [40]. A fluorescently labeled oligonucleotide probe is 15–25 base pairs (bp) in length, which can be generated on an automated synthesizer [42]. According to our experience, it is recommended to use the published rRNA-targeted oligonucleotide probes that have successfully detected the related microorganism(s) under a laser confocal microscope. Although some oligonucleotide probes have been used to obtain high FISH signal intensities in previously described microorganisms, it may not be the most suitable probes in another species, especially for those species that have a high mutation rate among different hosts (e.g., *Hodgkinia* in cicadas). Notably, it is necessary to check the identities of the rRNA-targeted oligonucleotide probe and 16S rRNA/18S rRNA gene sequence of the targeted microorganism(s) by the alignment of the probe sequence(s) and rRNA-targeted gene sequence(s) in the BLAST searches under the Nucleotide-BLAST program (Figure S2), which is vital to the accessibility of the oligonucleotide probe to the targeted sites.

It has been revealed that unlabeled oligonucleotides (helpers) binding to the adjacent probe target sites can open inaccessible rRNA regions for FISH with oligonucleotide probes [43]. Additionally, heat shock can denature the 16S rRNA/18S rRNA of related microorganism(s), thereby increasing the accessibility of fluorescent probes to rRNA-targeted sites. In our present study, it has been clearly shown that the fluorescence signal intensity of YLS was significantly increased by the addition of unlabeled helper sequences into a hybridization buffer combined with 95 °C heat shock of the hybridization sections for 2 min before hybridization, whereas the fluorescence signal intensity of Sulcia was generally similar between the control groups and treatment groups (Figure 6). The YLS fluorescence signal intensity of the control groups/treatment groups was approximately 1:2.95 in *K. caelatata* and 1:5.58 in *Ta*. sp., respectively (Figure 6), demonstrating that the fluorescence signal intensity of YLS can be enhanced by the addition of unlabeled helper sequences and 95 °C heat shock, whereas there was no obvious enhancement of the signal intensity of *Sulcia*. It is necessary to search for the optimal conditions of each targeted microbe under certain situations, and the combination of the most optimal hybridization conditions for each microbe seems to be the best choice for the detection of two or more microbes in a FISH experiment.

A previous study based on transmission and fluorescence microscopy showed that *Cardinium* sp. and YLS can be transovarially transmitted to the offspring in the leafhopper *Scaphoideus titanus*, but the FISH signal intensity of YLS in the fat bodies of *Sc. titanus* is quite weak [44]. It has been shown that *Sulcia* distributes in the digestive and excretory organs besides the bacteriomes and gonads in the cicada *Su. yangi* based on Illumina sequencing and fluorescence microscopy [28]. The distribution pattern(s) of the symbionts in cicadas and other insects merit a detailed investigation based on integrated methods. including the molecular sequencing, histological microscopy, and modified FISH technique, which would provide accurate information about the distribution of related symbionts. 

## 4. Materials and Methods

### 4.1. Samples Collection and Tissues Dissection

During the cicada emergence period, adults of *Ta*. sp. and *K. caelatata* were captured in the Huoditang Experimental Forest Station (33°26′ N, 108°26′ E; Ningshan County, Shaanxi Province, China) in the Qinling Mountains from middle-July to late-August of 2016–2022. *Ta*. sp. and *K. caelatata* were identified based on morphology and DNA barcode (*COI*). Specimens were subsequently transferred to the laboratory for dissection. The detailed dissection procedure was the same as previously described [17]. The freshly dissected samples were immediately preserved in a −80 °C freezer for genomic DNA extraction or fixed in 100% ethanol or 2.5% glutaraldehyde at 4 °C overnight for histological and fluorescence microscopy. Representative specimens were deposited in the Entomological Museum, Northwest A&F University (NWAFU), China.

### 4.2. DNA Extraction and IIIumina High-Throughput Sequencing of Bacterial 16S rRNA and Fungal ITS Gene

For Illumina high-throughput sequencing, three biological replicates were used for each sample. Extracted genome DNA of the bacterial 16S ribosomal RNA (rRNA) and fungal internal transcribed spacer (ITS1) were amplified using the published universal primers 515F (5-GTGCCAGCMGCCGCGGTAA-3) and 806R (5-GGACTACVSGGGTATCTAAT-3) [45] and ITS1_F_KYO2 (5-TAGAGGAAGTAAAAGTCGTAA-3) and ITS86R (5-TTCAAAGATTCGATGATTCAC-3) [46], respectively. The amplified PCR products were analyzed using 1% agarose gel electrophoresis and then purified with a Universal DNA Purification Kit (BioTeke, Beijing, China). Purified PCR products were determined by the concentration and quality using QuantiFlour^TM^ (Promega, Madison, USA) and subsequently sent to the sequence using an Illumina NovaSeq platform (Personal Biotechnology Co., Ltd., Shanghai, China) according to the standard protocols.

Low-quality raw sequences were checked and then filtered from the raw Illumina sequencing data [47]. The high-quality sequences imported into the Quantitative Insights Into Microbial Ecology version 2 (QIIME2) pipeline (http://qiime2.org, accessed on 18 January 2022) were processed to generate amplicon sequence variants (ASVs) via DADA2 (http://github.com/benjjneb/data2, accessed on 18 January 2022) based on 100% sequence similarity [48,49,50]. The taxonomic classification of ASVs was performed using QIIME2 through assigning against the SILVA databases, along with blasting the NCBI database manually with BLAST searches [51,52]. 

### 4.3. Diagnostic PCR Analyses of Dominant Symbionts in Different Tissues

Diagnostic PCR amplification was performed to confirm the presence of *Sulcia* and YLS in the salivary glands, gut tissues, reproductive organs, fat bodies, and bacteriomes. The PCR primers used in this study have been listed in Table S3. PCR cycle conditions used for the amplification of *Sulcia* and YLS were as previously described [7,9]. The PCR products were determined by 1% agarose gel electrophoresis and purified with a Universal DNA Purification Kit (BioTeke, Beijing, China). Representative PCR products were sent for sequencing at Tsingke Biological Technology Co., Ltd. (Beijing, China). The 16S rRNA gene sequences of *Sulcia* and 18S rRNA gene sequences of YLS were used as queries in BLAST searches through the NCBI GenBank nucleotide database.

### 4.4. Histological Microscopy Revealing the Distribution of Sulcia and YLS in Different Tissues of Cicadas

The salivary glands, filter chamber, conical segment, midgut, hindgut, reproductive organs (i.e., testes and ovaries), bacteriomes, and fat bodies were fixed in 2.5% glutaraldehyde at 4 °C overnight. The fixed samples were rinsed with 0.1 M phosphate-buffered saline (PBS, pH 7.2) for five times and post-fixed with 1% osmium tetroxide (OsO4) at 4 °C for 1.5 h. The samples were rinsed with 0.1 M PBS for six times and dehydrated in a graded ethanol series. The dehydrated samples were infiltrated with three mixtures of ethanol and LR White (3:1 for 2 h, 1:1 for 6 h, and 1:3 for 12 h) and pure LR White for 24 h twice. The samples were eventually embedded in pure LR White and polymerized at 60 °C for 48 h. Semithin sections (1 μM) were cut with a glass knife, stained with 1% methylene blue, and mounted in a neutral balsam (Servicebio, Wuhan, China) with the 24 × 40 mm microscope glass coverslips. Semithin sections were deposited in the Entomological Museum, Northwest A&F University (NWAFU), China.

### 4.5. Fluorescence In Situ Hybridization Confirming the Distribution of Sulcia and YLS in Different Tissues of Cicadas

The salivary glands, gut tissues, reproductive organs, bacteriomes, and fat bodies were fixed in 4% paraformaldehyde at 4 °C overnight; dehydrated in a graded ethanol series (75% for 4 h, 85% for 2 h, 90% for 2 h, 95% for 1 h, and 100% for 30 min twice); cleared four times in xylene for 2 h; and, finally, infiltrated with melted paraffin. Paraffin blocks were sectioned to 4 μM, and thin sections were used for histological or fluorescence microscopy. Paraffin sections used for histological microscopy were stained with hematoxylin and eosin, viz., HE staining. The detailed procedures of HE staining is provided in the Supplementary Materials (Figure S3), which has been successfully used for the staining of tissue sections and obtained high-quality images in other insects, including ants, beetles, honeybees, and butterflies. Notably, air-dried sections were immediately mounted in a neutral balsam, which can prevent the tissues oxidated after exposure to the air. The histological sections were deposited in the Entomological Museum, Northwest A&F University (NWAFU), China.

FISH experiments were conducted to further confirm the distribution of YLS and *Sulcia* in the salivary gland, gut tissues, reproductive organs, fat bodies, and bacteriomes of *Ta*. sp. and *K. caelatata*. The probe sequences and unlabeled helper sequences are listed in Table 1. The unlabeled helper sequences were thought to increase the accessibility of fluorescently labeled oligonucleotide probes to the targeted sites of related genes, thereby enhancing the hybridization signal intensity of related microorganism(s) [40,43]. We provide a schematic diagram (Figure S4A) to show the target sites of probe sequences and adjacent unlabeled helper sequences. A detailed hybridization procedure is provided in the Supplementary Materials (Figure S4B). Briefly, each slide was carried out in a final volume of 25 μL hybridization buffer contained 2.5× SSC (1× SSC is 0.15 M NaCl plus 0.015 M sodium citrate), 12.5% dextran sulfate, 0.25% bovine serum albumin (BSA), and fluorescently labeled probes (200 nM). The detailed components of the hybridization buffer used in this study are provided in Table 2. Hybridization was conducted in a 37 °C humidified chamber overnight. A negative control was done using no probe and only one symbiont-targeted probe staining to check the specificity of the hybridization.

We also compared the fluorescence signal intensity of *Sulcia* and YLS based on different treatments, aiming to explore whether the fluorescence signal intensity of these symbionts can be significantly enhanced by the addition of unlabeled helper sequences into the hybridization buffer combined with hybridization sections in 95 °C heat shock for 2 min before hybridization in the FISH experiments. The heat shock (i.e., 95 °C for 2 min) of the hybridization sections is thought to denaturate the rRNA of related microorganism(s), thereby increasing the accessibility of the fluorescent probes to rRNA-targeted sites [53]. Slides were observed and imaged under an Olympus FV 3000 IX inverted laser scanning confocal microscope (Olympus, Tokyo, Japan). Specifically, hybridization sections based on different treatments were imaged with the same parameters for the same symbiont within a cicada species. The fluorescence signal intensity of the hybridization sections was measured using ImageJ software. For each treatment, we usually measured at least 30 distinctly symbiont-harbored areas of the hybridization sections. We performed a nonparametric test (Kruskal–Wallis test) to determine the differences in the fluorescence signal intensities of hybridization sections based on different treatments.

### 4.6. Phylogenetic Relationships of Sulcia and YLS in Cicadas

The primers for the amplification of mitochondrial gene (i.e., *COI*, *COII*, *Cytb*) were the same as previously described (24). Multiple alignments of the nucleotide sequences of both cicada and their obligate symbionts were conducted using the online MAFFT program (https://mafft.cbrc.jp/alignment/server/index.html, accessed on 28 July 2022). Subsequently, the gappy columns at the beginning and end of the aligned sequences were edited manually to remove the gap-containing sites and ambiguously aligned sites using BioEdit software [54]. The phylogenetic trees of the 16S rRNA gene sequences of *Sulcia* and 18S rRNA gene sequences, together with four additional fungal nuclear gene sequences (*28S* + *Rpb1* + *Rbp2* + *EF1-α*) of the YLS obtained from the two cicada species and other representative cicada species, were reconstructed using maximum-likelihood (ML) using PhyloSuite version 1.2.2 [55] and Bayesian inference (BI) with the program MrBayes version 3.1.2 [56]. The phylogenetic trees of the representative cicada species were reconstructed based on combined mitochondrial gene sequences (*COI* + *COII* + *Cytb*) using maximum-likelihood and Bayesian inference. 

## 5. Conclusions

The results revealed that *Sulcia* was harbored in the bacteriomes, while YLS occupied the fat bodies of host cicadas. Both *Sulcia* and YLS can be transovarially transmitted between cicada generations, which formed a “symbiont ball” in the mature oocytes of the ovaries. *Sulcia* of *K. caelatata* shows the highest sequence similarity to that of *Me. iwasakii*, *Me. Kuroiwae,* and *Me. oshimensis*, which supports the mergence of Sinosenini with Dundubiini. The phylogenetic trees of both *Sulcia* and host cicadas show that Leptopsaltriini is not a monopyphyletic group. The phylogenetic trees of YLS and allied *Ophiocordyceps* suggest an evolutionary replacement of YLS in *Ta*. sp. from an *Ophiocordyceps* fungus to another *Ophiocordyceps* fungus, indicating complex evolutionary trajectories of YLS/*Ophiocordyceps* exist in different host species. We discovered that modification through the addition of helpers and heat shock can greatly enhance the FISH signal intensity of YLS, which may provide novel insights into the enhancement of the hybridization signal intensity of other symbiont(s) in the FISH experiments.

## Figures and Tables

**Table 1:** Probes used for the fluorescence in situ hybridization of *Sulcia* and YLS of two cicada species.

Probe Name	Fluorophore	Primer Sequence (5′–3′)	References
*Sulcia*	CY3	CCACACATTCCAGTTACTCC	[53]
*Sulcia*-Lhelper	unlabelled	GTTCTGTGTGATCTCTATGCATTTCACCGCT	
*Sulcia*-Rhelper	unlabelled	CCTCACTCTAGTTTATCAGTATCAATAGCACTT	
YLS	CY5	CCTGCCTGGAGCACTCT	[9]
YLS-Lhelper	unlabelled	CTAATGTATTCGAGCAT	This study
YLS-Rhelper	unlabelled	TTTTTCAAAGTAAAAGTCCCGT	

**Table 2:** The detailed hybridization solution used in this study.

Hybridization Solution	Final Concentration	Volume (100 μL)
25% dextran sulfate	10%	40 μL
10% bovine serum albumin	0.25%	2.5 μL
20 × SSC	2.5×	12.5 μL
ssDNA (200 ng/mL)	10 ng/μL	5 μL
*Sulcia* probe (2 μM)	200 nM	10 μL
*Sulcia*-Lhelper (100 μM)	2 μM	2 μL
*Sulcia*-Rhelper (100 μM)	2 μM	2 μL
YLS probe (2 μM)	200 nM	10 μL
YLS-Lhelper (100 μM)	2 μM	2 μL
YLS-Rhelper (100 μM)	2 μM	2 μL
diH_2_O	/	12 μL

## Data Availability

All sequences obtained in this study have been deposited in the NCBI nucleotide database under the accession numbers: OQ167977–OQ167978, OQ214195–OQ214196, OQ216585–OQ216588, and OQ222170–OQ222171. The Illumina data of bacteria symbionts obtained in this study have been deposited in GenBank under the accession number SRP321061. The Illumina data of fungal symbionts obtained in this study have been deposited in the Genome Sequence Archive (Genomics, Proteomics & Bioinformatics 2021) in the National Genomics Data Center (Nucleic Acids Res 2022), China National Center for Bioinformation/Beijing Institute of Genomics, and Chinese Academy of Sciences (accession number: PRJCA014276) that are publicly accessible at https://ngdc.cncb.ac.cn/gsa (accessed on 15 January 2023).

## Supplementary Materials

The following supporting information can be downloaded at: https://www.mdpi.com/article/10.3390/ijms24032434/s1.
