# Supplementary material for: A Study on Symbiotic Systems of Cicadas Provides New Insights into Distribution of Microbial Symbionts and Improves Fluorescence In Situ Hybridization Technique
Source: Int J Mol Sci. 2023 Jan 26;24(3):2434. doi: 10.3390/ijms24032434 (PMC9917331; doi:10.3390/ijms24032434)
Supplement: Supplementary file 1 [file ijms-24-02434-s001.zip › ijms-2150989-supplementary.pdf]

Supplemental materials

Table S1. Cicada samples used for diagnostic PCR amplification in this study

Species	Symbiont	Numbers of positive individuals/total numbers of individuals tested								
		SG	FC	CS	MG	HG	TE	OV	FB	BA
Karenia caelatata	*Sulcia*	0/12	0/12	0/12	0/12	0/12	0/6	6/6	0/12	12/12
	YLS	0/12	0/12	0/12	0/12	0/12	0/6	6/6	12/12	0/12
Tanna sp.	*Sulcia*	0/10	0/10	0/10	0/10	0/10	0/5	5/5	0/10	10/10
	YLS	0/10	0/10	0/10	0/10	0/10	0/5	5/5	10/10	0/10

Abbreviations: SG, salivary glands; FC, filter chamber; CS, conical segment; MG, midgut; HG, hindgut; TE, testes; OV, ovaries; FB, fat bodies; BA, bacteriomes.

Table S2. NCBI blast results for the *Sulcia* and yeast-like fungal symbiont obtained from *Karenia caelatata* and *Tanna* sp.

**Symbionts**	**Query coverage**	**Representative sequences in GenBank**	**Percent identity (%)**
Sulcia of *Tanna* sp.	100%	*Sulcia* of *Tanna japonensis* (LC370573.1)	99.40%
	100%	*Sulcia* of *Terpnosia vacua* (LC370693.1)	98.96%
	100%	*Sulcia* of *Te. nigricosta* (LC370570.1)	98.81%
Sulcia of *Karenia caelatata*	100%	*Sulcia* of *Meimunna iwasakii* (LC370740.1)	99.93%
	100%	*Sulcia* of *Me. kuroiwae* (LC370734.1)	99.93%
	100%	*Sulcia* of *Me. oshimensis* (LC370730.1)	99.85%
YLS of *Tanna* sp.	99%	YLS of *Mogannia minuta* (LC370999.1)	95.38%
	99%	YLS of *Euterpnosia chibensis* (LC370887.1)	95.11%
	99%	YLS of *Ta. japonensis* (LC370811.1)	93.49%
YLS of *Karenia caelatata*	94%	YLS of *Me. oshimensis* (LC370947.1)	92.95%
	93%	YLS of *Me. opalifera* (LC371018.1)	90.78%
	94%	YLS of *Ta. japonensis* (LC370901.1)	92.77%

Table S3. The primers for amplification of dominant symbionts in this study

Target	Primer name	Primer sequence (5'–3')	References
Sulcia (16S rRNA)	10_CFB_FF	AGAGTTTGATCATGGCTCAGGATG	Moran et al., 2005
	1515_R	GTACGGCTACCTTGTTACGACTTAG	
YLS (18S rRNA)	Fng18S_82F	GAAACTGCGAATGGCT	Matsuura et al., 2018
	NS4	CTTCCGTCAATTCCTTTAAG	
YLS (18S rRNA)	Fng18S_82F	GAAACTGCGAATGGCT	Matsuura et al., 2018
	Fng18S_1067R	TMTCGTAAGGTGCCGA	
YLS (18S rRNA)	Hyp325F	GTTTGGGTAGTGGCCAAAC	Matsuura et al., 2018
	Hyp760R	CCTGCCTGGAGCACTCT	
YLS (18S rRNA)	CFS2-CS	TTCAACGAGGAAYCCCTA	Matsuura et al., 2018
	LSU_CS-1R	GCTTCACTCGCCGTTAC	
YLS (28S rRNA)	ITS_CS-1F	CATCGAATCTTTGAACGC	Matsuura et al., 2018
	NLB1_CS	CTTTACCTCATAAAACTGAGNTCG	
YLS (RPB1)	RPB1-OhpF1	GTCTWCCACCCGGGCTT	Matsuura et al., 2018
	RPB1-OhpR1	GGCRATGTCGTTGTCCAT	
YLS (RPB2)	RPB2-OphF1	YAAGAAGCGYCTCGACCT	Matsuura et al., 2018
	RPB2-OphR2	GGCAGACTGGTACGTGTT	
YLS (Ef1α)	Ef1α-OphF1	CCGGCCACCGTGACTTCAT	Matsuura et al., 2018
	Ef1α-Oph2218R	ATGACACCGACGGCRMCGGTYTG	

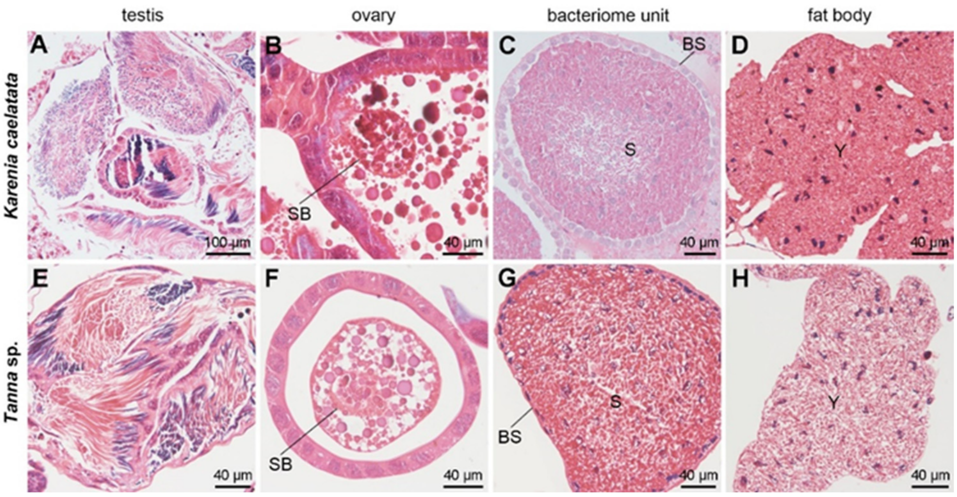

Figure S1. Histological microscopy showing the distribution of *Sulcia* and YLS in the testes, ovaries, bacteriomes and fat bodies of *Karenia caelatata* and *Tanna* sp. Abbreviations: BS, bacteriome sheath; S, *Sulcia*; Y, yeast-like fungal symbiont.

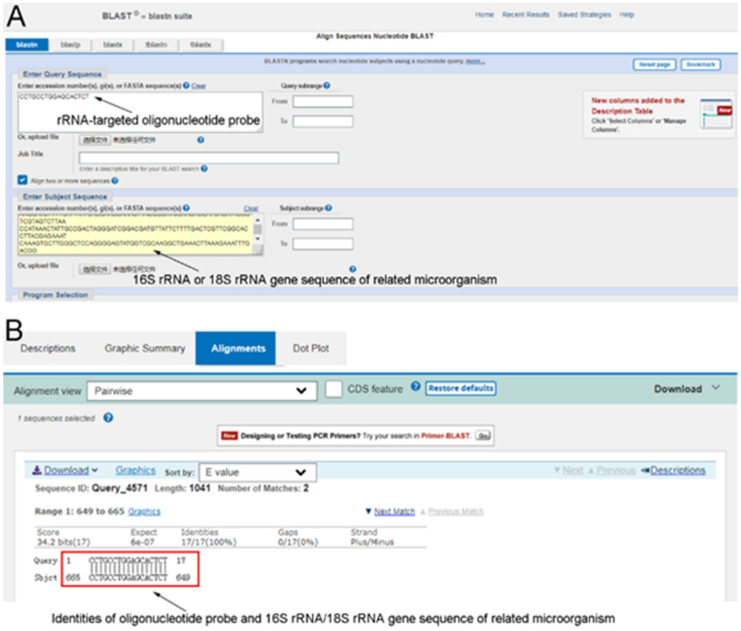

Figure S2. The identities of rRNA-targeted oligonucleotide probe and 16S rRNA/18S rRNA gene sequence of targeted microorganisms.

Solution	Time	Temperature
Xylene	20 min	room temperature
Xylene	20 min	room temperature
100% ethanol	10 min	room temperature
100% ethanol	10 min	room temperature
90% ethanol	5 min	room temperature
80% ethanol	5 min	room temperature
70% ethanol	5 min	room temperature
Double-distilled water	5 min	room temperature
Hematoxylin	8 min	room temperature
Double-distilled water	30 s	room temperature
Eosin	10 min	room temperature
Double-distilled water	30 s	room temperature

Figure S3. The detailed protocol for the paraffin sections stained with hematoxylin and eosin.

A

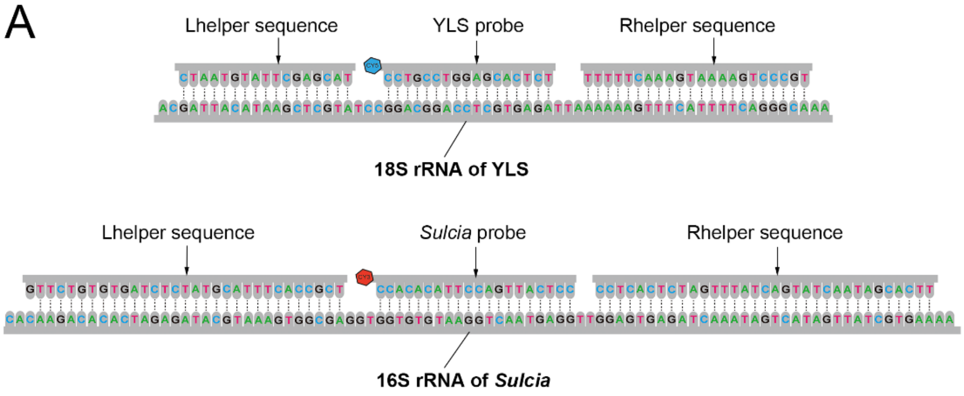

B

Solution	Time	Temperature
Xylene	20 min	room temperature
↓ Xylene	20 min	room temperature
↓ 100% ethanol	10 min	room temperature
↓ 100% ethanol	10 min	room temperature
↓ 90% ethanol	5 min	room temperature
↓ 80% ethanol	5 min	room temperature
↓ 70% ethanol	5 min	room temperature
↓ Double-distilled water	5 min	room temperature
↓ Hybridization buffer	8-12 h	37 ℃
↓ 2xSSC	~1 h	37 ℃
↓ Double-distilled water	5 min	room temperature
↓ DAPI solution	~3 min	room temperature
↓ Double-distilled water	2 min	room temperature

Figure S4. Schematic diagram showing the target sites of probe sequences and adjacent unlabeled helper sequences and the detailed protocol using for the hybridization procedure.
